# Gefitinib Inhibits Bleomycin-Induced Pulmonary Fibrosis via Alleviating the Oxidative Damage in Mice

**DOI:** 10.1155/2018/8249693

**Published:** 2018-04-12

**Authors:** Li Li, Lin Cai, Linxin Zheng, Yujie Hu, Weifeng Yuan, Zhenhui Guo, Weifeng Li

**Affiliations:** ^1^Department of Respiratory Medicine, General Hospital of Guangzhou Military Command of PLA, Guangzhou, Guangdong, China; ^2^Guangdong Food and Drug Vocational College, Guangzhou, Guangdong, China; ^3^Department of Respiratory Medicine, 458 Hospital of PLA, Guangzhou, Guangdong, China; ^4^MICU, Guangdong Provincial Key Laboratory of Geriatric Infection and Organ Function Support, General Hospital of Guangzhou Military Command of PLA, Guangzhou, Guangdong, China

## Abstract

Pulmonary fibrosis (PF) is a life-threatening interstitial lung disease. In this study, we tried to reveal the model of action between high-mobility group box 1 (HMGB1) and *α*-smooth muscle actin (*α*-SMA) and the protective role of gefitinib in pulmonary fibrosis induced by the administration of bleomycin aerosol in mice. For the mechanism study, lung tissues were harvested two weeks after modeling to detect the coexpression of HMGB1 and *α*-SMA by immunohistochemistry and immunofluorescence staining. Protein-DNA interactions were analyzed using a pulldown assay to study the relationship between HMGB1 and *α*-SMA. For the gefitinib treatment study, the mice were divided into three groups: phosphate-buffered saline (PBS) control group, PBS-treated PF group, and gefitinib-treated PF group. Gavage of gefitinib or PBS (20 mg/kg/day) was performed after bleomycin treatment for two weeks until the mice were sacrificed. Lung and blood samples were collected to assess the histological changes, oxidative stress, and expression of NOXs, HMGB1, EGFR, MAPKs, AP-1, and NF-*κ*B to determine the curative effect and related molecular mechanisms. The results revealed the high coexpression of *α*-SMA and HMGB1 in some interstitial cells in the fibrotic lung. The DNA-protein pulldown analysis proved that HMGB34367 acted as a novel transcriptional factor for the *α*-SMA promoter and participated in the eventual development of pulmonary fibrosis. Second, gefitinib could significantly decrease lung fibrotic changes and the level of MDA and recover the T-AOC level. Meanwhile, gefitinib could also reduce the NOX1/2/4, HMGB1, p-EGFR, p-ERK, p-JNK, p-P38, p-NF-*κ*B, p-c-Jun, and p-c-Fos expression levels in fibrotic lungs. The present study suggested that gefitinib could alleviate lung fibrosis through the HMGB1/NOXs-ROS/EGFR-MAPKs-AP-1/NF-*κ*B signal in bleomycin-induced pulmonary fibrosis.

## 1. Introduction

Idiopathic pulmonary fibrosis (IPF) is a chronic interstitial lung disease with high lethality, a fatal prognosis, and a lack of effective medical therapies [1, 2]. The pathological characteristics of IPF mainly include the disruption of the pulmonary parenchymal matrix and replacement with fibrotic tissues [3, 4]. The development of IPF results in declining lung function and eventual respiratory failure, with a median survival of only 2-3 years following diagnosis [5]. Traditional drugs only have marginal effects against this disease [6]. Details of the mechanisms involved in IPF are also not well established, and there is an extreme lack of effective therapies for IPF.

One possible mechanism involved in IPF is the activation of *α*-smooth muscle actin (*α*-SMA). Higher expression of *α*-SMA is accompanied by more collagen deposition, indicating more severe pulmonary fibrosis (PF) [7]. Understanding the mechanism responsible for *α*-SMA activation is a potential way to determine the fibrotic pathogenesis. High-mobility group box 1 (HMGB1) is a highly conserved DNA-shepherding protein, which could translocate to the cytoplasm as well as the extracellular space during cell activation, injury, or death [8]. It has been demonstrated that HMGB1 could promote the expression of *α*-SMA in fibrotic diseases [9]. The detailed mode of action between HMGB1 and *α*-SMA in pulmonary fibrosis, however, has not yet been fully interpreted. Another mechanism involved in pulmonary fibrosis is the activation of the epidermal growth factor receptor (EGFR). EGFR has been found to be upregulated in the lung tissues of patients and rodents with pulmonary fibrosis [10–12]. Gefitinib, an EGFR-tyrosine kinase inhibitor (EGFR-TKI), has the ability to inhibit fibroblast proliferation, therefore lessening the collagen and extracellular matrix (ECM) deposition in pulmonary fibrosis [13, 14]. However, the concrete mechanism behind this inhibition has not yet been clearly determined. Oxidative stress is another key pathological process in the development of pulmonary fibrosis [15]. Previous studies have shown that bleomycin administration could increase oxidative stress by increasing the production of nitric-oxide synthase and NADPH oxidase (NOX) through the downstream phosphorylation expression of mitogen-activated protein kinases (MAPKs) [16]. Inhibition of oxidative stress and enhancement of antioxidative ability could alleviate pulmonary fibrosis [17].

Since the potential influence and mechanism of gefitinib on pulmonary fibrosis have not been fully studied, the aim of the present research was (1) to detect the model of action between HMGB1 and *α*-SMA in pulmonary fibrosis and (2) to study the mechanism and resulting effect of gefitinib in the treatment of pulmonary fibrosis.

## 2. Materials and Methods

### 2.1. Drug Sources

Bleomycin hydrochloride (BLM) was purchased from Zhejiang Haizheng Pharmaceutical Co. Ltd. and gefitinib was purchased from AstraZeneca (United States). Other chemicals and reagents were obtained from standard commercial sources.

### 2.2. Model of Bleomycin-Induced Pulmonary Fibrosis in Mice

**Figure 1:**
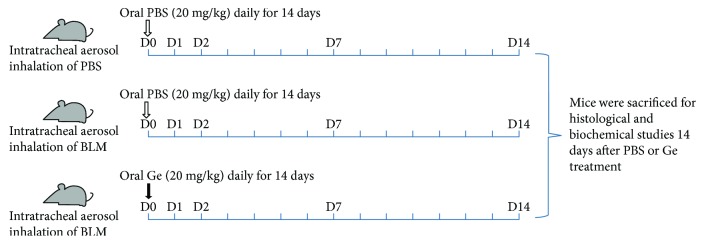
The experimental procedure of the present study.

SPF female KM mice (20–25 g) were purchased from the animal center of Southern Medical University (Guangzhou, China). Pulmonary fibrosis was induced by intratracheal aerosol inhalation of 3 U/kg of bleomycin dissolved in 0.10 mL of saline using MicroSprayer atomization devices. Phosphate-buffered saline (PBS) control mice (*n* = 6, PBS group) received the same volume of intratracheal aerosol inhalation of PBS. The gefitinib-treated mice (*n* = 6, BLM + Ge group) were treated with gefitinib by gavage at a dose of 20 mg/kg/day dissolved in 0.20 mL saline after bleomycin administration. The PBS-treated mice (*n* = 6, BLM group) received the same volume of PBS by gavage after bleomycin administration. All mice in the three groups were sacrificed two weeks after treatment, and lung and blood samples were processed separately for histological and biochemical studies. The animal experiment was approved by the institutional committee of animal care. The detailed experimental procedure was presented in Figure 1.

### 2.3. Histological Studies

#### 2.3.1. Hematoxylin and Eosin and Masson's Trichrome Staining

For histological examination, the lungs were fixed in 10% buffered formalin and stained with hematoxylin and eosin (H&E) and Masson's trichrome. Histologic grading was performed by three experienced pathologists using a blinded semiquantitative scoring system. The severity of pulmonary inflammation and fibrosis was scored according to the methods described by Mikawa et al. [18] and Ashcroft et al. [19], respectively.

#### 2.3.2. Immunohistochemistry (IHC)

Immunohistochemistry staining was performed to identify the expression and location of *α*-SMA and HMGB1 in the fibrotic lungs. Antibodies used in the study were anti-*α*-SMA (1 : 100) and anti-HMGB1 (1 : 200) antibodies (Santa Cruz Biotechnology Inc., CA) diluted in PBS. Slides were deparaffinized and treated with 3% H_2_O_2_ in H_2_O to quench endogenous peroxidase activity. *α*-SMA and HMGB1 staining was performed at 4°C overnight, followed by exposure to anti-mouse secondary antibody for 60 min. Diaminobenzidine (DAB) (Maxim-Bio, Fuzhou, China) was used as the chromogen. Microscopic observation was performed using a Leica DM LB2 microscope equipped with a digital camera.

#### 2.3.3. Immunofluorescence Staining

For immunofluorescence staining of *α*-SMA and HMGB1, the sections were pretreated with proteinase K and then blocked with 10% normal donkey serum diluted in PBS containing 0.1% Triton. Sections were then incubated with the polyclonal rabbit anti-*α*-SMA (1 : 200) and anti-HMGB1 (1 : 300) antibodies (Santa Cruz Biotechnology Inc., CA) diluted in PBS. Tissue sections were washed and subsequently incubated with Alexa fluorophore 488 nm donkey anti-rabbit antibody at 1 : 500 in PBS for 90 min at room temperature and counterstained with 4′,6-diamidino-2-phenylindole (DAPI). Images were acquired using an inverted Leica CTR 6000 fluorescence microscope and were merged using a Leica Application Suite Advanced Fluorescence software (Leica Microsystems (UK) Ltd., Milton Keynes, UK).

### 2.4. DNA-Protein Pulldown Analysis

A total of 3.8 kb of double-stranded DNA fragments corresponding to the mouse *α*-SMA promoter fragment −1070 to +2582, including the first exon and part of the first intron (GenBank: U63129 and M57409), was amplified from the VSMP8 plasmid by PCR. The plasmid was a generous gift from Professor Art Strauch (Dorothy M. Davis Heart and Lung Research Institute, Columbus, US). The forward primer 5′-AGCCGTGGGAGCGTGAGT-3′ was linked with a biotin molecule at the N terminal; the reverse primer was 5′-AGACAGCGAGCGAGAAGC-3′. Next, 50 *μ*g of biotinylated DNA fragments were ligated on the surface of 50 *μ*L of streptavidin-coated magnetic beads from a kilobaseBINDER kit (Dynal, Finland) to form DNA-bead complexes according to the manufacturer's protocol [20]. Pulmonary nuclear extracts from BLM- or PBS-treated lung tissues were dialyzed in PBS and diluted to 3*μ*g/*μ*L. Next, 30 *μ*g of proteins were added to 250 *μ*L of a DNA-protein binding buffer (20 mM Hepes, 1 mM EDTA, 10 mM (NH_4_)SO_4_, 1 mM DTT, 0.2% Tween20, 30 mM KCl, 50 ng/*μ*L poly(d(IC)), 5 ng/*μ*L poly L-lysine, and Ph 7.6) mixed with DNA affinity beads and incubated in a roller at room temperature for 30 min. After being subjected to magnetic separation, the supernatant was discarded, and the beads were washed three times by resuspension in 500 *μ*L binding buffer. Proteins binding with DNA affinity beads were eluted with 20 *μ*L of 0.25 M KCl solution. All the operations mentioned above were in accordance with the protocol of the DNA-binding protein purification kit (Roche, Germany), and samples from the PBS and BLM mice were studied simultaneously. The eluted proteins were then subjected to SDS-PAGE electrophoresis and EMBL silver staining to show the lung nuclear proteins binding with the *α*-SMA promoter *in vitro*. The differentially displayed protein bands were determined by comparison with protein grams from BLM- and PBS-treated samples and were cut out from the gel with a sterile knife for further liquid chromatography-mass spectrometry (LC-MS/MS) analysis by the Beijing Huada Jierui Biotechnology Co. Ltd. (Beijing, China).

### 2.5. RNA Preparation and Reverse Transcriptase Polymerase Chain Reaction (RT-PCR)

RNA was extracted from the left lung lobes of mice from all three groups using the TRIzol Reagent (Invitrogen, US) according to the manufacturer's recommendations. The lung tissues including HMGB34367, *α*-SMA, NOX1/2/4, and *β*-actin mRNA transcripts were measured by RT-PCR. PCR products were size fractionated in 1.5% agarose gels and visualized by ethidium bromide staining. Cycling conditions were (1) 94°C for 5 minutes; (2) 30 cycles at 94°C for 40 seconds, 55°C for 40 seconds, and 72°C for 40 seconds; and (3) a final extension step at 72°C for 10 minutes. The PCR primers were presented as follows: *α*-SMA, forward primer 5′-ACCCAGATTATGTTTGAGACC-3′ and reverse primer 5′-CCGTCAGGCAGTTCGTAG-3′; HMGB34367, forward primer 5′-ATGGGCAAAGGAGATCCTA-3′ and reverse primer 5′-CCTCATCATCTTCCTCTTC-3′; NOX1, forward primer 5′-TGGCATCCCTTCACTCTGA-3′ and reverse primer 5′-GGCACGCTGGAATTTGTAC-3′; NOX2, forward primer 5′-CCCTCCTATGACTTGGAAATG-3′ and reverse primer 5′-TCCGTCCAGTCTCCCACAATA-3′; NOX4, forward primer 5′-AGACAAATGTAGACACTCACC-3′ and reverse primer 5′-CACAATAAAGGCACAAAGGT-3′; and *β*-actin, forward primer 5′-AGGGAAATCGTGCGTGACATCAAA-3′ and reverse primer 5′-ACTCATCGTACTCCTGCTTGCTGA-3′.

### 2.6. Dihydroethidium (DHE) Fluorescence Measurement

The ROS levels in the lungs were assessed by DHE fluorescence. The lung tissues were stored in ethanol-dry ice at −80°C. Serial lung sections (5 *μ*m thickness) were performed and incubated in DHE (10 mmol/L, 30 min, 37°C). The lung tissue fluorescence (adopt excitation at 490 nm, emission at 610 nm) was observed by using a fluorescence microscope.

### 2.7. Measurement of MDA and T-AOC

The malonaldehyde (MDA) and total antioxidant capacity (T-AOC) levels in the serum and lung tissues were detected by using commercial activity assay kits purchased from the Nanjing Jiancheng Bioengineering Institute.

### 2.8. Immunoprecipitation and Tyrosine Phosphorylation Assay

Lung proteins were extracted using a RIPA lysate. Equal amounts of proteins were subjected to 8% SDS-PAGE and separated proteins were electrophoretically transferred to polyvinylidene difluoride membranes. The blot was blocked with 5% nonfat dried milk, incubated overnight with anti-NOX1/2/4, anti-HMGB1, anti-EGFR, anti-ERK/JNK/P38, anti-NF-*κ*B/c-Jun/c-Fos, anti-phosphor-EGFR, anti-phosphor-ERK/JNK/P38, anti-phosphor-NF-*κ*B/c-Jun/c-Fos, and anti-*β*-actin antibody (Santa Cruz Biotechnology Inc., CA), and treated with rabbit anti-mice IgG conjugated with alkaline phosphatase. The protein blot was detected with ECL and the gray scale was analyzed using a Gel-Pro Analyzer system.

### 2.9. Statistical Analysis

GraphPad Prism 5.0 software (version 5.0, GraphPad Software Inc., La Jolla, CA, USA) was used for statistical analysis. All the data were expressed as the mean ± standard deviation (mean ± SD). Student's *t*-test (2 groups) and (or) one-way ANOVA (multiple groups) was used to detect differences. *P* values less than 0.05 were considered significant.

## 3. Results

### 3.1. Expression and Location of *α*-SMA and HMGB1 in Bleomycin-Induced Pulmonary Fibrosis

**Figure 2:**
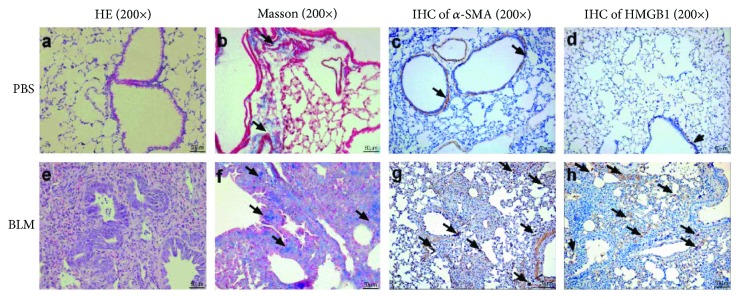
*α*-SMA and HMGB1 were highly expressed in the lung tissues affected by pulmonary fibrosis. Lung sections from mice were collected on day 14 after intratracheal bleomycin (BLM) or PBS administration and were stained with H&E, Masson's trichrome, and immunohistochemical stains (magnification 200x).

**Figure 3:**
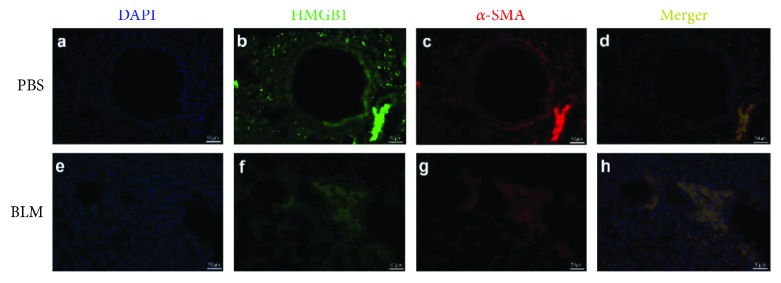
HMGB1 and *α*-SMA were coexpressed in some interstitial cells. Immunofluorescence staining for high-mobility group box 1 protein (HMGB1) (green) and *α*-smooth muscle actin (*α*-SMA) (red) and nuclei staining with DAPI (blue) were performed in fibrotic lung tissues. Yellow fluorescence staining (merged) indicated that some interstitial cells positively costained with HMGB1 and *α*-SMA (magnification 400x).

H&E and Masson's trichrome staining were adopted to examine the accuracy and efficiency of the pulmonary fibrosis model. H&E results demonstrated that BLM administration induced focal fibrotic lesions mainly in the subpleural regions with thickened or thickening interalveolar septa (Figures 2(a) and 2(e)). A similar result was observed by Masson's trichrome staining (Figures 2(b) and 2(f)). IHC results revealed that *α*-SMA and HMGB1 were highly expressed in the BLM-treated lung tissues. The interstitial cells surrounding the bronchioles of the BLM-treated lungs were positively stained by *α*-SMA (Figures 2(c) and 2(g)) and HMGB1 (Figures 2(d) and 2(h)), especially in the cells surrounding the terminal bronchioles. The immunofluorescence staining revealed the same colocalization of *α*-SMA and HMGB1 in some interstitial cells, which was in accordance with the IHC results (Figure 3).

### 3.2. HMGB34367 Increased Its Assembly with *α*-SMA in Pulmonary Fibrosis Conditions

**Figure 4:**
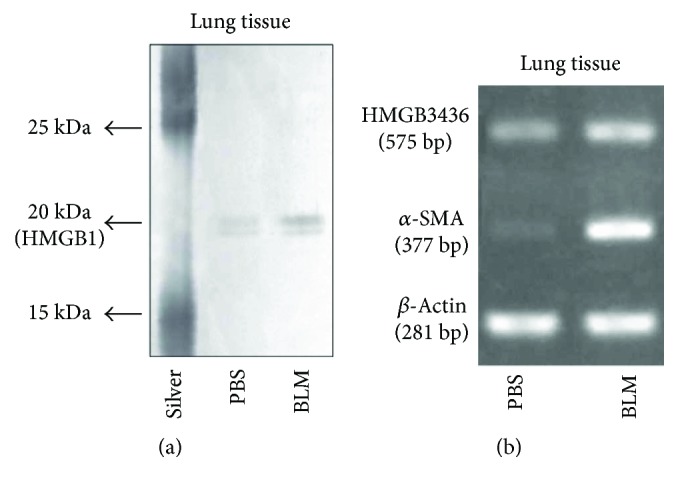
HMGB34367 was involved in direct regulation of *α*-SMA expression and was highly expressed in the lung tissues affected by pulmonary fibrosis. Lung tissues were collected 2 weeks after bleomycin (BLM) administration. Pulmonary nuclei were extracted from BLM- or PBS-treated lung tissue. (a) A DNA-nuclear protein pulldown method was used to detect the binding of nuclear proteins to the *α*-smooth muscle actin (*α*-SMA) promoter. A 20 kDa protein (HMGB34367) enlarged its binding amount with an *α*-SMA promoter under BLM conditions. (b) The mRNA expressions of *α*-SMA and HMGB34367 were increased in lung homogenates after BLM exposure.

To discriminate protein members composing the transcriptional complex in the activation of the *α*-SMA promoter, we developed a system to purify these binding proteins. The results showed that there was a characteristic increase in a 20 kDa protein in the BLM group of mice when compared with the PBS group (Figure 4(a). HMGB34367 was most likely to increase its assembly with *α*-SMA in pulmonary fibrosis conditions compared with physiological conditions. Next, we examined the mRNA expression of HMGB34367 and *α*-SMA in the BLM-treated lungs. RT-PCR analysis showed that the transcriptional levels of *α*-SMA and HMGB34367 were remarkably increased after BLM treatment (Figure 4(b)).

### 3.3. Gefitinib Decreased Pulmonary Fibrosis Induced by Bleomycin

**Figure 5:**
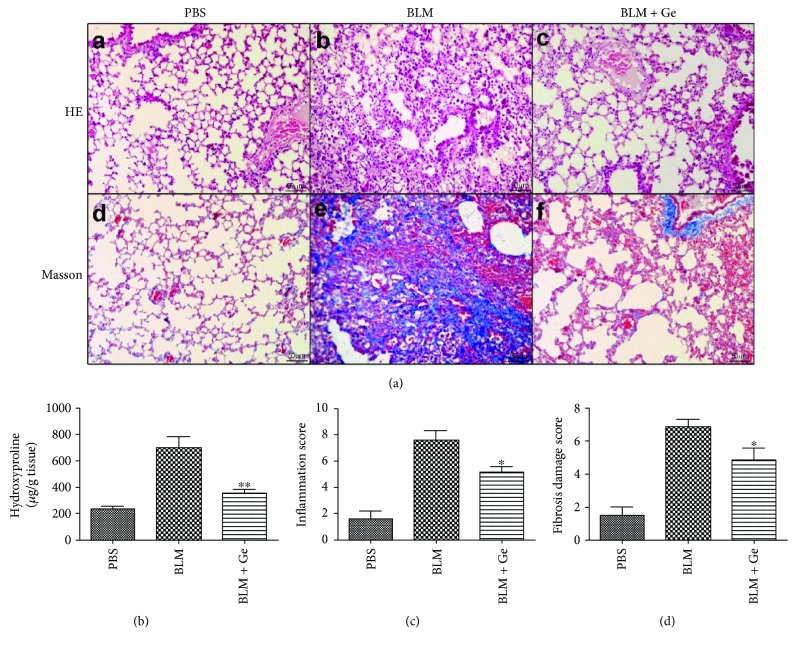
Gefitinib treatment decreased the severity of pulmonary fibrosis. Lung tissues from gefitinib- (Ge-) treated and PBS-treated pulmonary fibrosis mice were collected two weeks after bleomycin (BLM) administration. (a) Pathologic findings of lung tissues stained by H&E and Masson's trichrome (magnification 200x). (b) Lung hydroxyproline levels were significantly decreased by gefitinib treatment. Semiquantitative scoring of the severities and extents of inflammation (b) and fibrosis (c) were reduced in the BLM + Ge group compared with the BLM group. All data were expressed as the mean ± SD, *n* = 6. ^∗^*P* < 0.05 versus BLM group, ^∗∗^*P* < 0.01 versus BLM group.

Lung tissues from the BLM group of mice showed prominent peribronchiolar and interstitial infiltration with inflammatory cells. Extensive cellular thickening of interalveolar septa, interstitial edema, increasing of interstitial cells with a fibroblastic appearance, and interstitial collagen deposition could be detected by the Masson's trichrome staining. Although multifocal parenchymal lesions were still present in lung tissues from the BLM + Ge group mice, the local consolidation was smaller than those in the BLM group. Less edema and collagen deposition, less septal widening, and fewer clusters of inflammatory cells were observed in the lungs from the BLM + Ge group (Figure 5(a)). The lung hydroxyproline (a marker of collagen deposition) levels were increased approximately 3-fold in the BLM group of mice compared with the PBS group (696.34 ± 87.21 *μ*g/g tissue versus 234.52 ± 21.67 *μ*g/g tissue, *P* < 0.001). Treatment with gefitinib significantly reduced the hydroxyproline level (351.28 ± 32.93 *μ*g/g tissue versus 696.34 ± 87.21 *μ*g/g tissue, *P* < 0.001) (Figure 5(b). Meanwhile, semiquantitative scoring of the inflammation (Figure 5(c)) and fibrosis (Figure 5(d)) showed reduced levels in the BLM + Ge group when compared with the BLM group.

### 3.4. Serum MDA and T-AOC Levels

**Figure 6:**
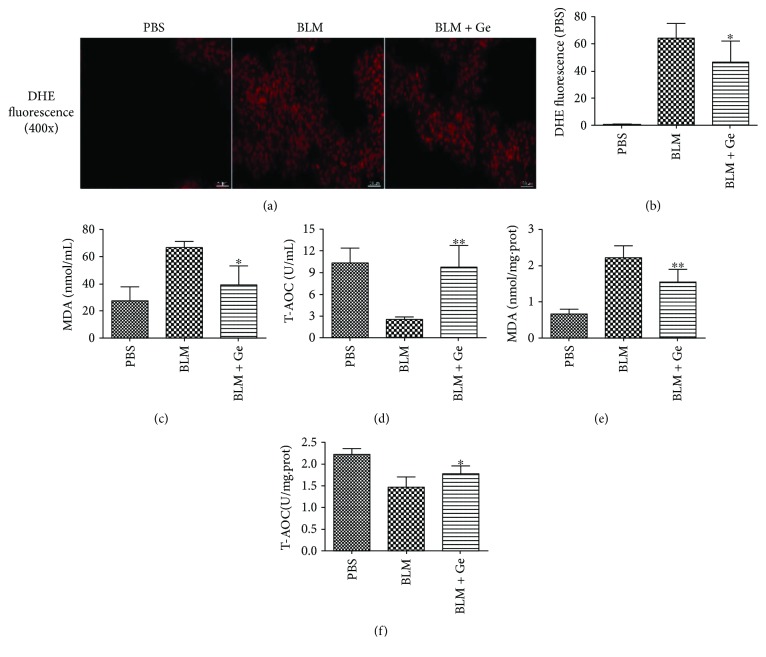
Gefitinib treatment alleviated the oxidative stress of pulmonary fibrosis. Lung tissues from gefitinib- (Ge-) treated and PBS-treated pulmonary fibrosis mice were collected two weeks after bleomycin (BLM) administration. (a) DHE fluorescence was used to detect ROS in the lung tissue (magnification 400x). (b) DHE fluorescence intensity was calculated. Gefitinib treatment significantly decreased serum (c) MDA and increased serum (d) T-AOC in mice. The levels of (e) MDA and (f) T-AOC in the lung tissues from the three groups showed the same results. All data were expressed as the mean ± SD, *n* = 6. ^∗^*P* < 0.05 versus BLM group, ^∗∗^*P* < 0.01 versus BLM group.

The imbalance in the oxidation-antioxidant activity is an important pathogenesis in pulmonary fibrosis. Previous reports have shown that excessive oxidative stress plays an important role in the pathogenesis of pulmonary fibrosis [15]. DHE fluorescence was conducted to measure ROS levels in lung tissues. A significant attenuation of fluorescence intensity was noted in the BLM + Ge group when compared with the BLM group (Figures 6(a) and 6(b)). MDA is the metabolite of lipid peroxidation, which could represent the degree of oxidative damage to histiocytes. T-AOC reflects the level of antioxidative capacity in mice. Bleomycin produces a significant increase in serum MDA level compared with the PBS group (66.70 ± 4.46 nmol/mL versus 26.92 ± 10.86 nmol/mL, *P* < 0.01), while treatment with gefitinib significantly reduced the MDA level (39.16 ± 14.15 nmol/mL versus 66.70 ± 4.46 nmol/mL, *P* < 0.05) (Figure 6(c)). The level of serum T-AOC had an inverse tendency compared with the level of MDA, and gefitinib treatment significantly inhibited the reduction of T-AOC after bleomycin administration (9.78 ± 2.94 U/mL versus 2.47 ± 0.44 U/mL, *P* < 0.01) (Figure 6(d)). The levels of MDA (Figure 6(e)) and T-AOC (Figure 6(f)) in the lung tissues also showed the same tendency. These results suggested that gefitinib could alleviate the excessive oxidative stress and enhance the antioxidative ability in the fibrotic lung.

### 3.5. Gefitinib Decreased the Gene and Protein Levels of NOX1/2/4 Induced by Bleomycin

**Figure 7:**
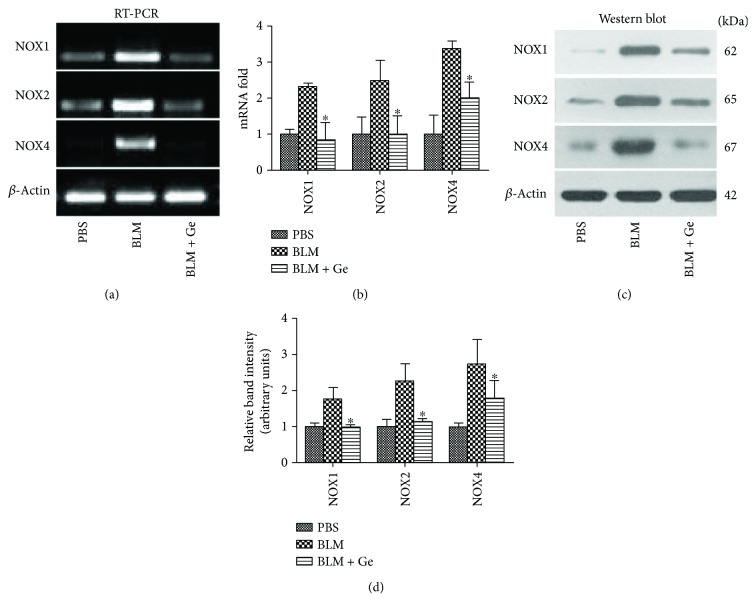
Gefitinib treatment decreased gene and protein expressions of NOX1/2/4 in the lung tissues affected by pulmonary fibrosis. Lung tissues from gefitinib- (Ge-) treated and PBS-treated pulmonary fibrosis mice were collected two weeks after bleomycin (BLM) administration. (a) The NOX1/2/4 gene expressions were identified by reverse transcriptase polymerase chain reaction (RT-PCR). (b) The relative mRNA folds were calculated. (c) The NOX1/2/4 protein expressions were identified by Western blot. (d) The relative band densities were calculated. The NOX1/2/4 gene and protein expressions in the lung tissues were significantly increased after BLM administration. However, gefitinib treatment significantly inhibited NOX1/2/4 expressions. All data were expressed as the mean ± SD, *n* = 6. ^∗^*P* < 0.05 versus BLM group.

Activation of NOX enzymes was the major source of ROS production in cells, and NOX1/2/4 were considered to play an essential role in the development of pulmonary fibrosis [21, 22]. In the present study, the lung tissue NOX1/2/4 gene and protein levels in mice exposed to bleomycin were all increased compared with the PBS group. Mice receiving gefitinib treatment after bleomycin administration showed a significant reduction in NOX1/2/4 gene (Figures 7(a) and 7(b)) and protein (Figures 7(c) and 7(d)) levels. These findings confirmed that NOX1/2/4 played an important role in the excessive ROS production in pulmonary fibrosis, and the high expressions could be blocked by gefitinib treatment.

### 3.6. Gefitinib Significantly Inhibited the HMGB1 Expression and Phosphorylation of EGFR-MAPK Signal Transduction

**Figure 8:**
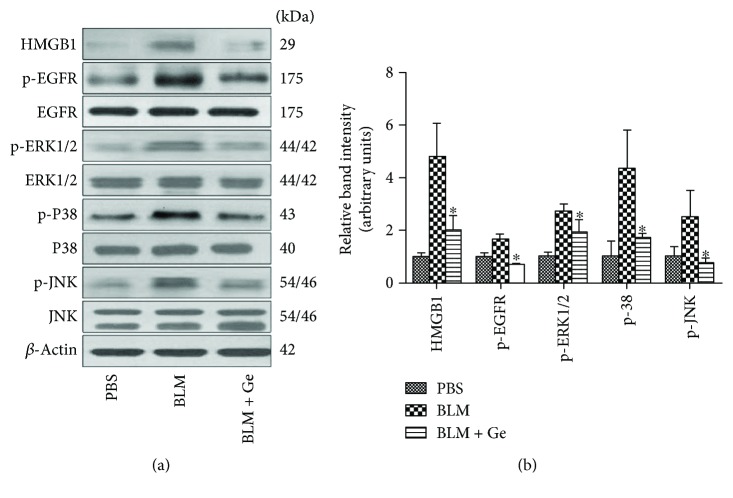
Gefitinib treatment decreased HMGB1 expression and phosphorylation expressions of EGFR and MAPKs in the lung tissues affected by pulmonary fibrosis. Lung tissues from gefitinib- (Ge-) treated and PBS-treated pulmonary fibrosis mice were collected two weeks after bleomycin (BLM) administration. (a) The expression of HMGB1, total, and phosphorylation of EGFR/ERK/P38/JNK in the lung tissues were detected by Western blot. (b) The relative band densities were calculated. Gefitinib treatment significantly inhibited the protein activation of HMGB1 and the phosphorylation of EGFR and MAPKs induced by BLM administration in mice. All data were expressed as the mean ± SD, *n* = 6. ^∗^*P* < 0.05 versus BLM group.

We measured the HMGB1, total, and phosphorylation expressions of EGFR and MAPKs in the fibrotic lung by Western blot. The results showed that gefitinib significantly inhibited HMGB1 expression, the phosphorylation of EGFR, and subsequently, the phosphorylation of ERK, P38, and JNK (Figure 8).

### 3.7. Gefitinib Decreased the Activations of AP-1 and NF-*κ*B

**Figure 9:**
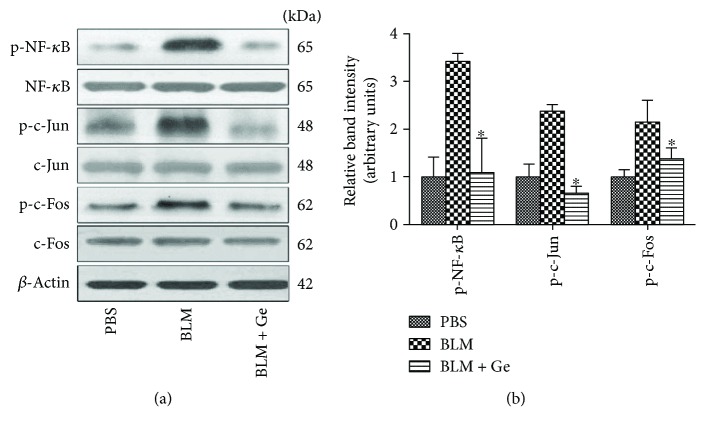
Gefitinib treatment decreased phosphorylation of NF-*κ*B, c-Jun, and c-Fos in the lung tissues affected by pulmonary fibrosis. Lung tissues from gefitinib- (Ge-) treated and PBS-treated pulmonary fibrosis mice were collected two weeks after bleomycin (BLM) administration. (a) The total phosphorylation of NF-*κ*B, c-Jun, and c-Fos in the lung tissues were detected by Western blot. (b) The relative band densities were calculated. Gefitinib treatment significantly inhibited the phosphorylation of NF-*κ*B, c-Jun, and c-Fos induced by BLM administration in mice. All data were expressed as the mean ± SD, *n* = 6. ^∗^*P* < 0.05 versus BLM group.

Previous studies showed that AP-1 and NF-*κ*B could be activated by the phosphorylation of ERK, JNK, and P38 [23]. Activation of AP-1 and NF-*κ*B could promote the production of proinflammatory factors and profibrotic factors in pulmonary fibrosis [24, 25]. The present study showed that administration of gefitinib did not influence the expressions of NF-*κ*B, c-Jun, and c-Fos in BLM-induced lung tissues. However, the phosphorylation of NF-*κ*B, c-Jun, and c-Fos were all significantly inhibited in accordance with the increased expression of MAPKs after gefitinib treatment (Figure 9).

## 4. Discussion

**Figure 10:**
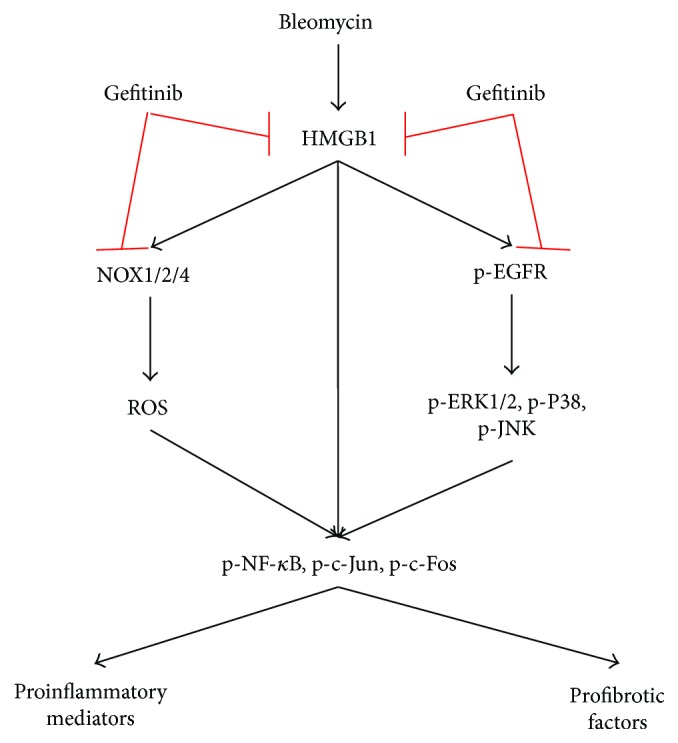
Schematic of gefitinib treatment in pulmonary fibrosis induced by bleomycin administration.

In the present study, we demonstrated the relationship between HMGB1 and *α*-SMA and the therapeutic effect of gefitinib in bleomycin-induced pulmonary fibrosis in mice. First, we proved that a member of the HMGB1 protein family (HMGB34367) could act as a novel transcriptional factor for the *α*-SMA promoter and participate in the process of pulmonary fibrosis. Second, we found that gefitinib was effective in reducing pulmonary fibrosis in mice, which might be associated with the HMGB1/NOXs-ROS/EGFR-MAPKs-AP-1/NF-*κ*B signal (Figure 10).

First, we concentrated on the role of HMGB1 in the development of pulmonary fibrosis. HMGB1 was highly expressed in the fibrotic lung tissues, and inhibition of HMGB1 could significantly alleviate lung injury in mice. Scientists mainly attributed the phenomenon to its role as an important inflammatory factor, and inflammation was a key pathological process in pulmonary fibrosis [26]. However, the detailed molecular mechanism of HMGB1 remained unknown. Immunohistochemistry and immunofluorescence results showed that HMGB1 was located in some interstitial cells in which *α*-SMA staining was also positive. RT-PCR analysis also showed that the mRNA expression of *α*-SMA was increased in parallel with the enhanced mRNA expression of HMGB34367 after BLM treatment. Moreover, we found that HMGB34367 was involved in the regulation of *α*-SMA expression by using a DNA-nuclear protein pulldown method. HMGB34367 was a 20 kDa protein belonging to a specific protein group, most of which had the same structure with HMGB1. The full-length sequence of HMGB34367 was identical to the 1 to 178 amino acid region of HMGB1 protein, which included two DNA binding domains, box A and box B, but lacked the 179–215 acidic C terminal tail region of HMGB1. HMGB1, a highly conserved nuclear protein, could stabilize nucleosomes and allow bending of DNA to facilitate some gene transcription [27]. Taken together, our results suggested that HMGB1 probably participated in the pulmonary fibrosis mainly by (i) digesting into different forms and facilitating *α*-SMA gene activation as a transcriptional activator and (ii) being secreted by activated monocytes and macrophages and behaving as an inflammatory cytokine.

Next, we studied the effect of gefitinib on pulmonary fibrosis. In fact, the potential of gefitinib to attenuate pulmonary fibrosis remained controversial. Hardie et al. demonstrated that gefitinib could apparently alleviate the pulmonary fibrosis in mice by decreasing EGFR expression and reversing the formative fibrosis in mice [14]. Subsequently, Ishii et al. found that all different oral doses (20 mg/kg, 90 mg/kg, and 200 mg/kg) of gefitinib could inhibit pulmonary fibrosis in mice, and large doses of gefitinib would not induce or aggravate pulmonary fibrosis [13]. However, Suzuki et al. and Li et al. showed that gefitinib treatment could markedly aggravate lung fibrosis in mice and rats [28, 29]. Moreover, severe lung interstitial disease associated with gefitinib treatment had been reported in some Japanese patients, implying that gefitinib might induce or aggravate this disease [30, 31]. In fact, there were several possible explanations for the different results. First, it might be related to the different breeds of mice or rats that had different responses to gefitinib because of their genetic characteristics. Second, the different doses of gefitinib were important factors causing the contradictory results. Higher doses of gefitinib could result in toxicity more than therapy. Third, the expression and activation of EGFR in different breeds of rodents and their responses to different doses of gefitinib had direct effects on the MAPK signals which could promote or inhibit pulmonary fibrosis. Fourth, the time of administration as well as the method of administration of gefitinib during the experiment also affected the results. Moreover, recent phase II gefitinib clinical trials in Japan announced that there were no differences in the incidence of pulmonary fibrosis between patients who used gefitinib and other chemotherapeutics, suggesting that gefitinib might not be the cause of pulmonary fibrosis [32]. In our previous series of studies, we found that gefitinib apparently inhibited lung collagen deposition and *α*-SMA expression induced by bleomycin in fibrotic mice [33, 34].

To elucidate the antifibrosis mechanism of gefitinib, we investigated oxidative and antioxidant levels in pulmonary fibrosis. Gefitinib completely inhibited the high concentration of MDA induced by bleomycin and recovered the T-AOC ability in pulmonary fibrotic mice. It was certainly well established that NOXs were the primary sources of ROS production that mediated various pulmonary diseases, including IPF and pulmonary hypertension [35, 36]. There were seven members of the NOX family found in mammals: NOX1, NOX2, NOX3, NOX4, NOX5, Duox1, and Duox2. Among them, NOX1, NOX2, and NOX4 had been shown to contribute to tissue fibrosis [37, 38]. NOX4 mRNA and protein expressions were upregulated in pulmonary fibroblasts from patients with IPF and correlated with *α*-SMA and procollagen I (*α*1) mRNAs [39]. NOX4-deficient mice were protected from bleomycin-induced pulmonary fibrosis through modulation of epithelial cell death *in vivo* and decreased TGF-*β*1-mediated ROS production and protection from apoptosis [40]. NOX1 and NOX2 were also reported to contribute to tissue fibrosis in nonpulmonary organ systems. HMGB1 was a proinflammatory factor that was also linked to oxidative stress [8]. The present study showed that gefitinib could also inhibit HMGB1 expression. However, how could this happen, and could gefitinib inhibit the HMGB1 and EFGR synchronously? In fact, HMGB1 could directly signal MAPKs to activate the NF-*κ*B pathway [41]; moreover, HMGB1 could also indirectly signal the MAPK pathway by EGFR activation [42]. Extensive evidence indicated that the MAPK pathway was the downstream signaling cascade of EGFR, which participated in the development of pulmonary fibrosis [43]. A previous study showed that gefitinib inhibited the expressions of collagen I and III mRNAs by blocking the transactivation of EGFR and the subsequent activation of ERK1/2 in kidney fibrosis [44]. In vascular smooth muscle cells, EGFR increased production of nitric-oxide synthase and NOX enzymes through the downstream phosphorylation expression of ERK and AKT [45]. Our present data implied that gefitinib inhibited pulmonary fibrosis *in vivo* through HMGB1/NOXs-ROS/EGFR-ERK/JNK/P38 pathways.

NF-*κ*B and AP-1 were the important transcription factors that were downstream of ROS and MAPKs [46]. The NF-*κ*B signal transduction pathway was an important pathway involved in inflammation, immunity, cell proliferation, and apoptosis [47]. Previous studies have indicated that in acute lung injury activation of NF-*κ*B could induce the production of proinflammatory mediators; contrarily, inhibiting the NF-*κ*B activation significantly alleviated lung injury [24, 48]. AP-1, as a redox-regulated transcription complex (composed of c-Fos and c-Jun), was activated by MAPK family members via enhancing their downstream transcription factors including Elk-1, c-Jun, ATF2, and CREB, which in turn regulated the expressions of c-Fos and c-Jun. AP-1 and MAPKs proteins could be activated and augmented cyclically [49]. AP-1 and NF-*κ*B could promote the production of proinflammatory mediators and profibrotic factors in pulmonary fibrosis. In the present study, AP-1 and NF-*κ*B participated in the development of pulmonary fibrosis, and this progress was regulated by the phosphorylation and expression of ROS, HMGB1, and MAPKs.

Although many experiments were performed to reveal the effect and mechanism of gefitinib on the development of pulmonary fibrosis, shortcomings also existed. First, it was an animal study, and more evidence from clinical trials and *in vitro* researches are urgently needed. Second, we did not perform knockout, knockdown, or overexpression studies, which were limited by our experimental conditions. We only used RT-PCR, histological examinations, and Western blot to study the detailed molecular mechanisms. In the future, *in vivo* studies utilizing related knockout or knockdown mice as well as *in vitro* studies using primary lung cells and fibroblasts should be launched to verify the mechanism.

## 5. Conclusions

In summary, we comprehensively investigated the role of the molecule HMGB1, the involvement of oxidation-antioxidant unbalance, and the related molecule transduction mechanism downstream of gefitinib treatment in bleomycin-induced pulmonary fibrosis. These results indicated that HMGB1 contributed to the pulmonary fibrosis and gefitinib attenuated bleomycin-induced collagen deposition and excessive oxidative stress, mainly by suppressing the HMGB1/NOXs-ROS/EGFR-MAPKs-AP-1/NF-*κ*B signal. The present findings have the potential to fundamentally advance our understanding of the molecular mechanism of pulmonary fibrosis and could have important implications in the design of novel and innovative therapeutic approaches targeting pulmonary fibrosis.
